# The effect of tropical cyclone on cognitive function in older adults: A longitudinal study from Thailand

**DOI:** 10.1093/geroni/igaf122.2283

**Published:** 2025-12-31

**Authors:** Fei Sun, Jin Ke, Phatchanun Vivarakanon

**Affiliations:** Michigan State University, East Lansing, Michigan, United States; Huazhong University of Science and Technology, Wuhan, Hubei, China (People’s Republic); Boromarajonani College of Nursing, Nakhon, Lampang, Thailand

## Abstract

In the context of climate change, tropical cyclones (TCs) represent an increasingly severe threat to human health, particularly among vulnerable populations such as older adults. This study investigated the impact of TC exposure on cognitive function among older adults in Thailand, focusing on specific cognitive domains and exploring potential underlying mechanisms. Data were obtained from two key sources: the Health, Aging, and Retirement in Thailand (HART) survey and the Emergency Events Database (EM-DAT). These datasets were integrated to assess both the short- and long-term effects of TC exposure using a fixed-effects model. Cognitive function was measured across three key dimensions: memory, calculation, and time orientation. Additionally, the study examined depression, hypertension, and social isolation as potential pathways through which TCs might affect cognitive health. The findings revealed that exposure to TCs had a significant and lasting negative impact on the calculation dimension of cognition, whereas the effects on memory and time orientation were either absent or short-lived. Specifically, individuals exposed to TCs demonstrated significantly lower calculation scores on the day of exposure, and this decline persisted for up to four years post-exposure. Further analyses suggested that depression, exacerbation of hypertension, and increased social isolation were contributing factors to this cognitive decline. These findings underscore the long-term cognitive risks associated with extreme weather events and highlight the need for targeted public health interventions. Policies and practices to mitigate the cognitive impact of TCs among older adults, particularly in climate-vulnerable regions are needed.

